# Super pathogens from environmental biotechnologies threaten global health

**DOI:** 10.1093/nsr/nwab110

**Published:** 2021-06-25

**Authors:** Yong Xiao, Feng Zhao, Josep Peñuelas, Qiansheng Huang, Yong-Guan Zhu

**Affiliations:** CAS Key Laboratory of Urban Pollutant Conversion, Institute of Urban Environment, Chinese Academy of Sciences, China; CAS Key Laboratory of Urban Pollutant Conversion, Institute of Urban Environment, Chinese Academy of Sciences, China; CSIC, Global Ecology Unit CREAF-CSIC-UAB, Spain; CREAF, Autonomous University of Barcelona, Spain; CAS Key Laboratory of Urban Environment and Health, Institute of Urban Environment, Chinese Academy of Sciences, China; CAS Key Laboratory of Urban Environment and Health, Institute of Urban Environment, Chinese Academy of Sciences, China; State Key Laboratory of Urban and Regional Ecology, Research Center for Eco-Environmental Sciences, Chinese Academy of Sciences, China

## Abstract

The incubation and release of super pathogens from environmental biotechnologies is an overlooked threat to global health. This perspective calls for collaboration between research community, industry and government to mitigate this growing risk.

Microbial pathogens are viruses, bacteria, fungi and protozoa infecting humans, animals or plants. Super pathogens (also known as superbugs) able to resist multiple antimicrobials and cause fatal infections have been increasingly considered an emerging threat to global health [1]. The COVID-19 pandemic has caused more than 3.8 million deaths as of 15 June 2021, reminding us that we should always be alert to the next pandemic caused by super pathogens. We know little, however, about the origin and transmission of super pathogens in the environment. Among the various potential sources of super pathogens, special attention should be paid to environmental biotechnologies (EBTs) for treating wastewater.

Human activities generate waste-water, with a global production of ∼330 km^3^/year [2], that contains heavy metals, carbohydrates, antimicrobials and pathogens polluting the environment and spreading diseases. Various methods have been developed to purify wastewater, but EBTs are the most common ones because they use microbes to degrade pollutants economically, i.e. at a cost of <0.1 US$/m^3^ municipal wastewater in China.

EBTs are valuable for purifying wastewater, but they carry a risk of incubating and releasing super pathogens. Millions of microbial species exist in EBTs, many of which are pathogens playing important roles in degrading pollutants [3]. EBTs, e.g. municipal wastewater-treatment plants, have been widely reported as hotspots of pathogens and antimicrobial resistance genes (ARGs) [4]. During long-term use of EBTs, pathogens can evolve to resist multiple pollutants, which unfortunately enhances their resistance to antimicrobials, because microbes use similar strategies to resist antimicrobials and toxic pollutants such as heavy metals [5]. Microbial genes can also be transferred between microbes [6], enabling pathogens to acquire new ARGs from others to become super pathogens. Furthermore, traditional disinfection can also increase the spread of ARGs between different microbes.

Fecal coliforms or *Escherichia coli* are widely used microbial indexes for the discharge of treated wastewater, e.g. 100–10 000 colony-forming units/L in some countries. The abundance of fecal coliforms in EBTs is low, e.g. <0.22% in most wastewater-treatment plants [3], suggesting that a substantial volume of other microbes, including super pathogens, is released to the natural environment and then spread around the world by human activities [7]. For example, super pathogens that produce New Delhi metallo-beta-lactamase 1 (NDM-1) conferring resistance to multiple antimicrobials are spread to rivers via effluent from wastewater-treatment plants lacking proper sanitation [8]. Super pathogens from EBTs can be transferred to humans mainly via accidental contact with reclaimed water and bioaerosols from EBTs and foods contaminated by EBT effluent. We therefore call for close cooperation between the research community, industry and government to reduce the threat to global health caused by super pathogens from EBTs (Fig. 1).

**Figure 1.**
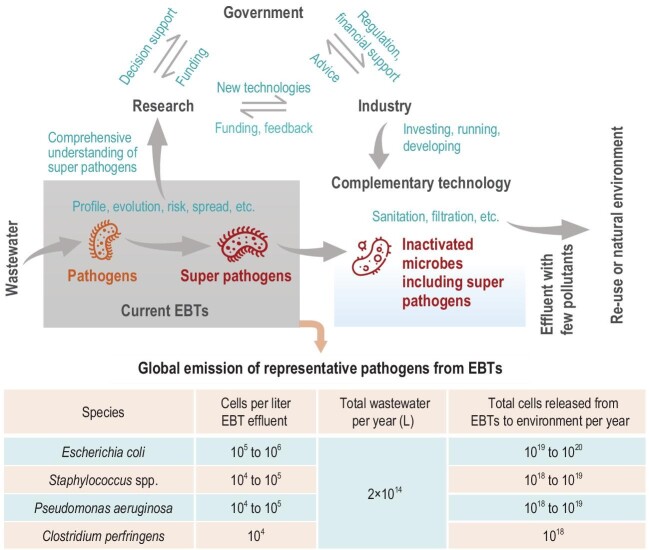
Collaboration between the research community, industry and government can contribute to therapeutic EBTs for a healthier world. EBTs for removing pollutants are producing and releasing other harmful pollutants, e.g. super pathogens. Huge numbers of pathogenic microbes are now being released from EBTs. See Supplementary Data for data sources.

‘Know yourself and know your enemy, and you will never be defeated’, as said in *Sunzi Bingfa (The Art of War)*. First, we must better understand the evolution of super pathogens in EBTs on a global scale. Many studies have investigated the dynamics of ARGs in the ecosystem, but little is known about super pathogens. Monitoring the emission of super pathogens from EBTs and tracking their fates in various environments is important for evaluating their risks to human health. Such research can inform the public with regard to avoiding environments and products contaminated by EBT effluent, and most importantly, can support governments to appropriately supervise the EBT industry. High-throughput sequencing of nucleic acids provides community-level information, but information about pathogens is lacking. Third-generation sequencing targeting single DNA molecules is a better alternative, and generates long sequence reads linking super pathogens with their ARGs or virulence genes.

Current EBTs mainly aim at removing traditional carbohydrates and nutrients instead of super pathogens, so substitutive or complementary technologies are needed to eliminate the release of super pathogens. For example, macrophyte-assisted vermifiltration can efficiently treat wastewater, and earthworms can consume and kill pathogens. Complementing current EBTs with sustainable disinfection processes, e.g. eBeam and nanobubble technologies, rather than replacing them with new technologies, however, is more cost-effective. eBeam technology, using electron beams for disinfection and pollutant degradation, was launched in a demonstration project in 2017 in Zhejiang, China. Nanobubble technology uses bubbles (<1000 nm in diameter) without chemicals to degrade pollutants and inactivate pathogens. Researchers from Harvard University, for example, created tiny, aerosolized water nanodroplets containing reactive oxygen species or other non-toxic, nature-inspired disinfectants to kill pathogens efficiently [9].

Governments play key roles in solving the problem of super pathogens by guiding the public, supervising industry and supporting research, but most governments have not yet recognized the public health threat of super pathogens from EBTs. The most efficient way for governments to solve the problem of super pathogens is to set stricter standards on microbial pathogens for EBT discharge. Setting standards, however, is extremely challenging, and requires a comprehensive understanding of the diversity, evolution, spread and risk of super pathogens. Complementary facilities can help current EBTs to meet stricter standards, which would substantially increase the cost of treating wastewater. Current EBTs for hospitals, livestock farms and pharmaceutical factories can easily incubate super pathogens and should be the highest priority for complementary disinfection.

Our world is a closely connected community, and microbes can be spread around the world by human activities [7], meaning all countries are required to act together to defeat super pathogens. Low-income countries lack funds to construct wastewater-treatment plants with new but expensive technologies, and cost-effective EBTs like vermifiltration are valuable options for them. High-income countries have complete wastewater-treatment systems, but they could better complement their current EBTs with sustainable disinfections like eBeam and nanobubble technologies. Intergovernmental cooperation, e.g. by high-income countries providing financial and technological aid to low-income countries, is crucial for preventing worldwide pandemics. High-income countries could also train researchers and engineers from low-income countries to protect the environment.

Untreated wastewater can exacerbate sanitation-related problems, which can create barriers to achieving the 2030 Sustainable Development Goals (SDGs) of the United Nations, such as good health and well-being (SDG3) and clean water and sanitation (SDG6). The world, however, is still rapidly urbanizing, which brings challenges to pollution reduction, the supply of fresh water and the safeguarding of health (e.g. by eliminating super pathogens). Though EBTs contribute to these SDGs, we need to recognize the dangers of super pathogens from EBTs. The outbreak of COVID-19 has indicated the potential spread of viruses by wastewater-treatment systems [10], but little has been done to eliminate the health threat of super pathogens from EBTs. A One Health systems approach (inter-connectivity of human, animal and ecosystem health) is needed for understanding microbial cycling between environment, animals and humans.

## Supplementary Material

nwab110_Supplemental_File

## References

[1] David S, Reuter S, Harris SR et al. Nat Microbiol 2019; 4: 1919–29. 10.1038/s41564-019-0492-8 31358985 PMC7244338

[2] Mateo-Sagasta J, Raschid-Sally L, Thebo A. In: Drechsel P, Qadir M, Wichelns D (eds.). *Wastewater: Economic Asset in an Urbanizing World*. Dordrecht: Springer Netherlands, 2015, 15–38.

[3] Wu L, Ning D, Zhang B et al. Nat Microbiol 2019; 4: 1183–95. 10.1038/s41564-019-0426-5 31086312

[4] Li J, Chen H, Bond PL et al. Water Res 2017; 123: 468–78. 10.1016/j.watres.2017.07.002 28689130

[5] Baker-Austin C, Wright MS, Stepanauskas R et al. Trends Microbiol 2006; 14: 176–82. 10.1016/j.tim.2006.02.006 16537105

[6] Partridge SR, Kwong SM, Firth N et al. Clin Microbiol Rev 2018; 31: e00088–17. 10.1128/CMR.00088-17 30068738 PMC6148190

[7] Zhu Y-G, Gillings M, Simonet P et al. Science 2017; 357: 1099–100. 10.1126/science.aao3007 28912233

[8] Yang F, Mao D, Zhou H et al. Environ Sci Technol Lett 2016; 3: 138–43.

[9] Vaze N, Pyrgiotakis G, McDevitt J et al. Nanomed Nanotechnol Biol Med 2019; 18: 234–42. 10.1016/j.foodcont.2018.09.037 PMC6588479 30904585

[10] Lodder W, de Roda Husman AM. Lancet Gastroenterol Hepatol 2020; 5: 533–4. 10.1016/S2468-1253(hskipz@20)30087-X 32246939 PMC7225404

