# Supplementary material for: Super pathogens from environmental biotechnologies threaten global health
Source: Natl Sci Rev. 2021 Jun 25;8(9):nwab110. doi: 10.1093/nsr/nwab110 (PMC8433084; doi:10.1093/nsr/nwab110)
Supplement: nwab110_Supplemental_File [file nwab110_supplemental_file.pdf]

Supplementary materials
for

Super pathogens from environmental biotechnologies threaten global health

Yong Xiao[1], Feng Zhao[1], Josep Peñuelas[2,3], Qiansheng Huang[4], Yong-Guan Zhu[*,4,5]

1, CAS Key Laboratory of Urban Pollutant Conversion, Institute of Urban Environment, Chinese Academy of Sciences, Xiamen 361021, China

2, CSIC, Global Ecology Unit CREAF-CSIC-UAB, Barcelona 08193, Spain

3, CREAF, Autonomous University of Barcelona, Barcelona 08193, Spain

4, CAS Key Laboratory of Urban Environment and Health, Institute of Urban Environment, Chinese Academy of Sciences, Xiamen 361021, China

5, State Key Laboratory of Urban and Regional Ecology, Research Center for Eco-environmental Sciences, Chinese Academy of Sciences, Beijing 100085, China

*Corresponding author. E-mail: ygzhu@iue.ac.cn.

This file includes:

Materials and Methods

Table S1

References

Materials and Methods

Using combinative keywords of *Escherichia coli*, *Staphylococcus aureus*, *Pseudomonas aeruginosa*, *Clostridium perfringens*, effluent, wastewater treatment plant, CFU, we retrieved reports describing quantitative investigation on the abundance and prevalence of the representative pathogens in effluent in wastewater treatment plant from Google Scholar. CFU is the abbreviation of colony forming unit. The detailed data is shown in Table S1. All the data are based on culture and plate count methods, using treated wastewater as inoculant. The cell concentrations in source papers reported in CFU/ml or CFU/100ml were uniformly converted to CFU/L. Due to that most wastewater treatment plants do not have disinfection before discharging treated wastewater, we used the data from wastewater treatment plants without disinfection. Javier reported a global wastewater production of about 330 km^3 per year, and 60 % of the produced municipal wastewater is treated in early 21st century [1], which meant that about 200 km^3 of municipal wastewater was treated each year using EMTs.

Table S1 Summary of culture data for representative microbes or microbial groups in effluent of wastewater treatment plant.

Species/Genus/ Group	EBT Type	Location	CFU / L	Disinfection treatment	Reference
Escherichia coli	Municipal wastewater treatment plant	Poznań, Polands	1.0×10^5 to 3.0×10^5	No disinfection	[2]
Escherichia coli	Municipal wastewater treatment plant	Durban, Kwazulu Natal Province, South Africa	1.0×10^3 to 2.0×10^3	Chlorination	[3]
Escherichia coli	Urban Wastewater Treatment Plant without industrial discharge	Georgia, USA	1.2×10^2	UV+chlorination	[4]
Escherichia coli	Urban wastewater treatment plant	Besançon, France	3.7×10^6	No disinfection	[5]
Escherichia coli	Urban wastewater treatment plant	Rouen, France	6.5×10^5	No disinfection	[6]
Escherichia coli	Urban Wastewater Treatment Plant receiving hospital wastewater	Olsztyn, Poland	2.7×10^6	No disinfection	[7]
Escherichia coli	Municipal wastewater treatment plant	Warszawa, Poland	3.2×10^4 to 1×10^5	No disinfection	[8]
Escherichia coli	Wastewater treatment plant	Hamburg, Germany	2.5×10^4	Chemical treatment	[9]
Escherichia coli	Municipal wastewater treatment plant	Pachuca, Hidalgo, Mexico	3.1×10^4	Disinfected by NaClO twice	[10]
Escherichia coli	Six sewage treatment plants	Ebro River basin, Spain	1×10^3 to 1×10^6	No disinfection but some with tertiary treatment	[11]

extended-spectrum β-lactamase-producing *Escherichia coli*	Urban wastewater treatment plant	Besançon, France	2.2×10^{4}	No disinfection	[5]
Staphylococcus spp.	Urban wastewater treatment plants	La Rioja region, Spain	8×10^{3}	No disinfection	[12]
Mannitol-positive staphylococci	Municipal wastewater treatment plant	Warszawa, Poland	1.5×10^{5}	No disinfection	[8]
Methicillin Resistant *Staphylococcus aureus*	Sewage treatment plant	The City of Thibodauxi, Louisiana, USA	5.4×10^{6}	UV light as a disinfecting agent	[13]
Staphylococcus aureus	Three sewage treatment plants	Sydney, Australia	1.7×10^{2}	No disinfection	[14]
Staphylococcus aureus	Six sewage treatment plants	Ebro River basin, Spain	1×10^{4} to 6×10^{5}	No disinfection but some with tertiary treatment	[11]
Pseudomonas aeruginosa	Three sewage treatment plants	Sydeny, Australia	2.2×10^{5}	No disinfection	[14]
Pseudomonas aeruginosa	Urban wastewater treatment plant	Besançon, France	1.1×10^{4}	No disinfection	[15]
Pseudomonas aeruginosa	Discharge site of sewage treatment plant	Peniche, Portugal	1.4×10^{3}	Not Applicable	[16]
Pseudomonas aeruginosa	Municipal wastewater treatment plant	Pachuca, Mexico	5×10^{2}	Disinfected by NaClO twice	[10]
Pseudomonas aeruginosa	Pilot plant treating light greywater	Haifa, Israel	1.2×10^{3}	UV disinfection	[17]
Pseudomonas spp.	Three sewage treatment plants	rural Alice, peri-urban Dimbaza, and urban East London, South Africa	1.08×10^{5} to 2.66×10^{5}	Chlorination	[18]

Pseudomonas spp.	Six sewage treatment plants	Ebro River basin, Spain	1×10^4 to 6×10^7	No disinfection but some with tertiary treatment	[11]
Pseudomonas spp.	Municipal wastewater treatment plants	Karlsruhe, Germany	8×10^6	No ozone disinfection	[19]
Pseudomonas spp.	Municipal wastewater treatment plants	Karlsruhe, Germany	8×10^2	Ozone disinfection	[19]
Clostridium perfringens	Three sewage treatment plants	Sydeny, Australia	6.8×10^5	No disinfection	[14]
Clostridium perfringens	Wastewater treatment plant	Hamburg, Germany	5.4×10^3 (MPN/L)	Chemical treatment	[9]
Clostridium perfringens	Sequencing batch biofilter granular reactor treating sewage	Bari, Italy	2.4×10^4	Biological disinfection	[20]
Clostridium perfringens	Sequencing batch biofilter granular reactor treating sewage	Bari, Italy	$1.4\text{-}2.1\times10^4$	UV radiation or peracetic acid disinfection	[20]
Clostridium perfringens	Activated sludge wastewater treatment plants	Bolivar, Australia	$1\text{-}2\times10^4$	No disinfection	[21]
Clostridium perfringens	Sewage treatment plants	Ljubljana, Slovenia	6.8×10^3	No disinfection	[22]

References:

1. Mateo-Sagasta, J., L. Raschid-Sally, and A. Thebo, *Global wastewater and sludge production, treatment and use*, in *Wastewater: Economic Asset in an Urbanizing World*, P. Drechsel, M. Qadir, and D. Wichelns, Editors. 2015, Springer Netherlands: Dordrecht. 15-38.
2. Koczura, R., et al., *Antimicrobial resistance of integron-harboring Escherichia coli isolates from clinical samples, wastewater treatment plant and river water.* Science of The Total Environment, 2012. **414**: 680-685.

3. Adegoke, A.A., et al., *Antibiogram and beta-lactamase genes among cefotaxime resistant E. coli from wastewater treatment plant.* Antimicrobial Resistance & Infection Control, 2020. **9**(1): 46.
4. Aslan, A., et al., *Presence of Antibiotic-Resistant Escherichia coli in Wastewater Treatment Plant Effluents Utilized as Water Reuse for Irrigation.* Water, 2018. **10**(6): 805.
5. Bréchet, C., et al., *Wastewater treatment plants release large amounts of extended-spectrum β-lactamase–producing Escherichia coli into the environment.* Clinical Infectious Diseases, 2014. **58**(12): 1658-1665.
6. Oberlé, K., et al., *Evidence for a Complex Relationship between Antibiotics and Antibiotic-Resistant Escherichia Coli: From Medical Center Patients to a Receiving Environment.* Environmental Science & Technology, 2012. **46**(3): 1859-1868.
7. Korzeniewska, E., A. Korzeniewska, and M. Harnisz, *Antibiotic resistant Escherichia coli in hospital and municipal sewage and their emission to the environment.* Ecotoxicology and Environmental Safety, 2013. **91**: 96-102.
8. Szyłak-Szydłowski, M., A. Kulig, and E. Miaśkiewicz-Pęska, *Seasonal changes in the concentrations of airborne bacteria emitted from a large wastewater treatment plant.* International Biodeterioration & Biodegradation, 2016. **115**: 11-16.
9. Ajonina, C., et al., *Microbial pathogens in wastewater treatment plants (WWTP) in Hamburg.* Journal of Toxicology and Environmental Health, Part A, 2015. **78**(6): 381-387.
10. Coronel-Olivares, C., et al., *Chlorine disinfection of Pseudomonas aeruginosa, total coliforms, Escherichia coli and Enterococcus faecalis: revisiting reclaimed water regulations.* Water Science and Technology, 2011. **64**(11): 2151-2157.
11. López, A., et al., *How does urban wastewater treatment affect the microbial quality of treated wastewater?* Process Safety and Environmental Protection, 2019. **130**: 22-30.
12. Gómez, P., et al., *Characterization of staphylococci in urban wastewater treatment plants in Spain, with detection of methicillin resistant Staphylococcus aureus ST398.* Environmental Pollution, 2016. **212**: 71-76.
13. Boopathy, R., *Presence of methicillin resistant Staphylococcus aureus (MRSA) in sewage treatment plant.* Bioresource Technology, 2017. **240**: 144-148.
14. Ashbolt, N.J., G.S. Grohmann, and C.S.W. Kueh, *Significance of specific bacterial pathogens in the assessment of polluted receiving waters*

of Sydney, Australia. Water Science and Technology, 1993. **27**(3-4): 449-452.
15. Slekovec, C., et al., *Tracking down antibiotic-resistant Pseudomonas aeruginosa isolates in a wastewater network.* PLOS ONE, 2012. **7**(12): e49300.
16. Januário, A.P., et al., *Faecal Indicator Bacteria and Pseudomonas aeruginosa in Marine Coastal Waters: Is there a Relationship?* Pathogens, 2020. **9**(1): 13.
17. Gilboa, Y. and E. Friedler, *UV disinfection of RBC-treated light greywater effluent: Kinetics, survival and regrowth of selected microorganisms.* Water Research, 2008. **42**(4): 1043-1050.
18. Odjadjare, E.E., et al., *Prevalence of Multiple Antibiotics Resistant (MAR) Pseudomonas Species in the Final Effluents of Three Municipal Wastewater Treatment Facilities in South Africa.* International Journal of Environmental Research and Public Health, 2012. **9**(6): 2092-2107.
19. Jäger, T., et al., *Live-dead discrimination analysis, qPCR assessment for opportunistic pathogens, and population analysis at ozone wastewater treatment plants.* Environmental Pollution, 2018. **232**: 571-579.
20. De Sanctis, M., et al., *Integration of an innovative biological treatment with physical or chemical disinfection for wastewater reuse.* Science of The Total Environment, 2016. **543**: 206-213.
21. Wen, Q., et al., *Fate of pathogenic microorganisms and indicators in secondary activated sludge wastewater treatment plants.* Journal of Environmental Management, 2009. **90**(3): 1442-1447.
22. Oprčkal, P., et al., *Critical evaluation of the use of different nanoscale zero-valent iron particles for the treatment of effluent water from a small biological wastewater treatment plant.* Chemical Engineering Journal, 2017. **321**: 20-30.
